# Genotyping-by-sequencing supports a genetic basis for wing reduction in an alpine New Zealand stonefly

**DOI:** 10.1038/s41598-018-34123-1

**Published:** 2018-11-02

**Authors:** Andrew J. Veale, Brodie J. Foster, Peter K. Dearden, Jonathan M. Waters

**Affiliations:** 1 Department of Zoology, University of Otago, Dunedin, 9016 New Zealand; 2 Department of Environmental and Animal Sciences, Unitec, Auckland, 1025 New Zealand; 3 Genomics Aotearoa and Department of Biochemistry, University of Otago, Dunedin, 9016 New Zealand

## Abstract

Wing polymorphism is a prominent feature of numerous insect groups, but the genomic basis for this diversity remains poorly understood. Wing reduction is a commonly observed trait in many species of stoneflies, particularly in cold or alpine environments. The widespread New Zealand stonefly *Zelandoperla fenestrata* species group (*Z*. *fenestrata*, *Z*. *tillyardi*, *Z*. *pennulata*) contains populations ranging from fully winged (macropterous) to vestigial-winged (micropterous), with the latter phenotype typically associated with high altitudes. The presence of flightless forms on numerous mountain ranges, separated by lowland fully winged populations, suggests wing reduction has occurred multiple times. We use Genotyping by Sequencing (GBS) to test for genetic differentiation between fully winged (n = 62) and vestigial-winged (n = 34) individuals, sampled from a sympatric population of distinct wing morphotypes, to test for a genetic basis for wing morphology. While we found no population genetic differentiation between these two morphotypes across 6,843 SNP loci, we did detect several outlier loci that strongly differentiated morphotypes across independent tests. These findings indicate that small regions of the genome are likely to be highly differentiated between morphotypes, suggesting a genetic basis for wing reduction. Our results provide a clear basis for ongoing genomic analysis to elucidate critical regulatory pathways for wing development in Pterygota.

## Introduction

Understanding the genetic basis of phenotypic variability not only illuminates active evolutionary processes occurring within species, but may also shed light on the evolution of different morphologies among species. Wing polymorphism has arisen in many insect orders, with variability in wing morphology particularly prominent in Hemiptera (true bugs), Coleoptera (beetles), Orthoptera (crickets and grasshoppers), and Plecoptera (stoneflies)^1–4^. Within these groups, flightless taxa are particularly common on islands, at high altitudes and high latitudes^1^. In some cases, however, the degree of wing development may vary between closely related species or even within a species. While referred to as “wing polymorphism”, such variation often consists of morphs that differ in all major aspects of flight capability (e.g. size of flight muscles, production of flight fuels), as well as many other aspects of physiology and reproduction. These polymorphisms may result from a variety of causes: alternate morphologies may be encoded by different genotypes (genetic polymorphism), induced by different environments (environmental polyphenism), or produced by variation in both genetic and environmental factors^5^. The degree of wing development can either be dimorphic with two alternative forms, or variation can exist along a spectrum.

There are many factors that influence the relative costs and benefits of flight in insects (reviewed by^2,6–8^). Wing reduction may confer an adaptive advantage when habitat stability is high, and when habitat complexity is low^9^. Habitat isolation may also promote flight loss, as the removal of flighted emigrants from habitat patches selects against this dispersal ability^1,7,10–12^. Specifically, in alpine environments high winds may sweep away fully winged individuals capable of flight^7,13–15^. Wing reduction has also been attributed to the high energy expenditure required in the production and maintenance of flight apparatus, with dispersal apparently traded off for life history traits such as fecundity^1,4,16–21^.

Stoneflies are of particular interest relating to the evolution of insect flight due to their early divergence within winged insects (Pterygota) and also because they exhibit multiple wing-powered locomotive behaviors, including sailing and surface skimming^22^. These methods of locomotion have even been proposed as models for the evolution of flight in insects^23–25^, and it has been suggested that stoneflies thus may exhibit an ancestral form of wing and flight development^22,26^. Many stonefly species have reduced wings, with four forms of wing-length polymorphism described: macropterism (fully winged or long-winged), brachypterism (short-winged), micropterism (vestigial-winged) and apterism (wingless)^27^. Even most fully winged stonefly taxa are typically considered to be weak flyers with limited dispersal ability^27–33^. There have been several studies of wing reduction in stoneflies e.g.^13,15,32,34–38^, with some suggesting a possible genetic basis for short wingedness e.g.^39^ but this hypothesis remains to be tested.

Over the last decade, high-throughput genetic sequencing, along with reduced representation genomic libraries^40^ have enabled the low-cost discovery and genotyping of thousands of genetic markers for non-model organisms, revolutionizing ecological, evolutionary and conservation genetics^41–43^. In particular, these advances have enabled the discovery of many candidate loci involved in specific phenotypic traits^44–46^. Such advances have been made either with quantitative trait loci (QTL) mapping using pedigree information, or through genome-wide association studies (GWAS) that identify non-random associations of alleles between loci and adaptive traits as a consequence of natural selection^47–49^.

The underlying bases for wing polymorphism have now been studied in several species of insects, revealing various environmental, developmental, and genetic controls, often with multiple developmental pathways and regulators e.g.^50^. For instance, the proximate endocrine processes that control wing development have been investigated in wing-polymorphic crickets (*Gryllus sp*.), showing Juvenile Hormone (JH) may regulate wing development in this species^5,51^, while in a planthopper (*Nilaparvata lugensor*), genes in the insulin-signaling pathway may regulate wing development^52,53^. The genes responsible for wing polymorphism have also recently been investigated in ants (*Pheidole morrisi*)^54^, salt marsh beetles (*Pogonus chalceus*)^55,56^ and pea aphids (*Acyrthosiphon pisum*)^57,58^. There are also known genes responsible for wing patterning and development in model organisms such as *Drosophila melanogaster*, which may be relevant to intra-specific wing polymorphism^59^. While genetic changes often underlie wing polymorphism, epigenetic changes have also been demonstrated between wing morphs in a planthopper (*Sogatella furcifera*)^60,61^.

**Figure 1:**
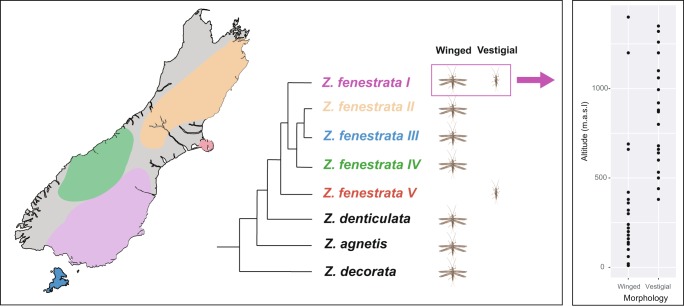
Map showing the distribution of each *Zelandoperla fenestrata* clade, along with the wing morphotypes present in each clade, and (at right) the altitudinal distribution of wing morphotypes for *Z*. *fenestrata* Clade 1 (data from McCulloch *et al*.^32^).

The New Zealand stonefly *Zelandoperla fenestrata* species group (*Z*. *fenestrata*, *Z*. *tillyardi*, *Z*. *pennulata*) contains populations that range from fully winged to vestigial-winged, with wing-reduced populations more prevalent in southern South Island, particularly at higher altitudes^62,63^. Under current taxonomy, micropterous individuals are classified as *Zelandoperla pennulata* (McLellan 1999), dark-colored individuals, including those implicated in the mimicry of another stonefly (*Austroperla cyrene*), are classified as *Zelandoperla tillyardi* (McLellan 1999), while the remaining light-colored fully winged individuals are classified as *Zelandoperla fenestrata* (Tillyard 1923). The three described species, however, appear to represent co-distributed color and wing-length polymorphisms rather than discrete evolutionary units, with the species group actually comprising five geographically discrete, deeply divergent clades (from 2–9% average divergence at COI)^32^. These five regional clades exhibit differing propensities to exhibit wing reduced populations (Fig. 1). Of the five clades of *Z*. *fenestrata* species group, Clade 1 is generally wing-dimorphic, with fully winged lowland populations and alpine-associated vestigial-winged populations, with a steep transition in wing morphology occurring at around 500 meters above sea level (m.a.s.l) (Fig. 1). In contrast, Clades 2–4 appear to be composed of only fully winged individuals, and Clade 5 is thought to be exclusively micropterous or apterous^63^. Given the level of divergence between clades, and the probable differences in developmental characteristics between them, these clades may represent different species; further study is warranted to reclassify this group. The apparent difference in propensity for wing reduction in different clades suggests the possibility of a genetic basis for wing reduction in these taxa. Furthermore, the presence of non-dispersive, flightless forms on multiple mountain ranges in *Z*. *fenestrata* Clade 1, separated by lowland winged populations, suggests wing reduction may have evolved multiple times in this lineage^32^. At finer spatial scales, recent genetic studies have shown phylogenetic divergence in wing-reduced populations of *Z*. *fenestrata* Clade 1 between adjacent mountain streams, highlighting the low dispersal ability of alpine populations and the possibility that each stream may have been colonized independently by winged lowland ancestors^64^. The specific mechanisms and genes behind wing development and polymorphism in *Z*. *fenestrata* remain unknown.

There are two (non-exclusive) hypotheses as to how *Z*. *fenestrata* Clade 1 lose their wings: (1) wing loss is genetically determined, or (2) wing loss is mediated by environmentally determined gene expression (i.e. polyphenism). Both of these hypotheses have received support from studies of other wing-dimorphic insects. Examples of taxa showing genetically determined wing dimorphism (Hypothesis 1) include several species of carabids and weevils^14,65,66^ where wing dimorphism is controlled by a single gene operating in a Mendelian fashion. Similarly, in field crickets^67^ maize leaf hoppers (*Cicadulina sp*.)^68^ and salt marsh beetles (*Pogonus chalceus*)^69^, wing polymorphism is genetically controlled but related to a complex interplay between many genes. However, in a situation more consistent with Hypothesis 2 (polyphenism), while wing morphology in *Gryllus* crickets can be controlled either by a single gene locus or a polygene complex, both can be regulated by the level of juvenile hormone (JH) – whereby if JH exceeds a threshold value during a critical developmental stage of the insect, wing development is suppressed^5,51,70^. Other environmental factors that can influence wing development include abiotic factors such as temperature^66^ and photoperiod^71^ as well as biotic factors such as food resources^66^ and population density^72^. Many of these environmental regulators of wing development also have a genetic component, for instance the fully winged morphotype of the red fire bug (*Pyrrhocoris apterus*) is determined by a recessive allele, whose penetrance depends on photoperiod and temperature^73^. Environmentally induced wing polyphenism in insects can also be transgenerational, with the level of the hormone ecdysone in the mother (regulated by population density) altering the expression of wing development in the offspring of the pea aphid (*Acyrthosiphon pisum*)^74^.

In this study, we use Genotyping By Sequencing (GBS) to test for genetic differentiation between wing morphotypes in *Z*. *fenestrata* Clade 1, and test for loci specifically associated with wing reduction. GBS analyzes a subset of the genome next to specific restriction sites, providing a near-random sample of SNP loci across the genome, some of which may be associated with differentially adaptive genes or regulatory regions^47–49^. As mentioned, *Z*. *fenestrata* Clade 1 represents a genetically distinctive lineage of the species group, with a propensity for alpine related wing-reduction, and it may be divergent enough from other clades to warrant reclassification to species or sub-species level. Surveys of *Z*. *fenestrata* Clade 1 morphotype distributions identified one stream (Black Jacks Creek) that exhibited an unusual pattern of substantial overlap between wing morphotype populations at a low altitude. By focusing our study on a single stream population that exhibits co-distributed extreme wing morphologies, we aim to examine genomic differentiation between morphotypes without the confounding factor of neutral genetic population structure or other environmental differences.

## Methods

### Sample collection

**Figure 2:**
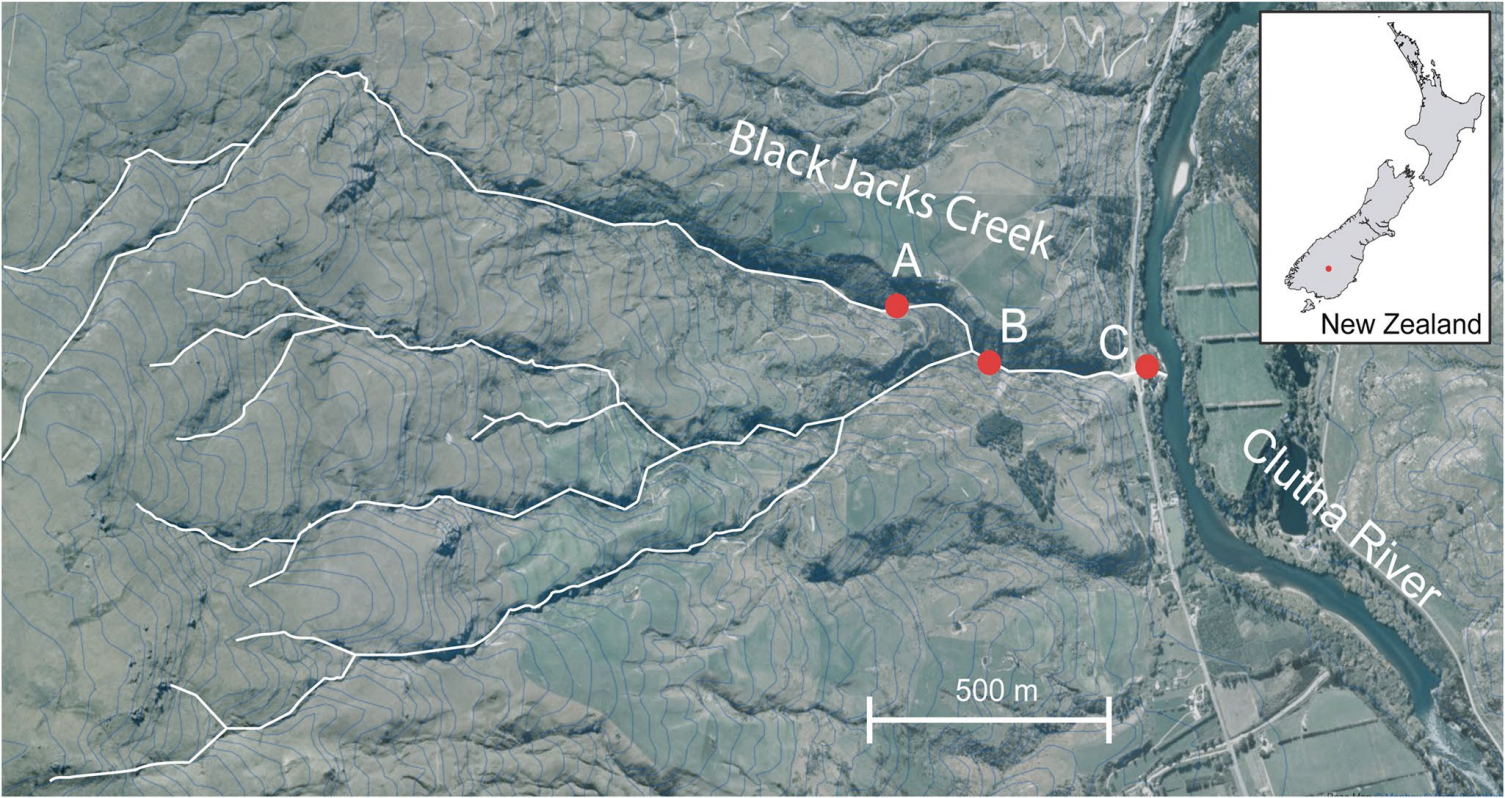
Map showing the sampling locations along Black Jacks Creek (A = 200 m.a.s.l, B = 130 m.a.s.l, C = 90 m.a.s.l). Map data sourced from the LINZ Data Service and licensed by Land Information New Zealand for re-use under the Creative Commons Attribution 4.0 International licence. https://data.linz.govt.nz/set/4702-nz-aerial-imagery/.

Sampling was conducted along Black Jacks Creek (−45.574559, 169.307399) on the Old Man Range, South Island, New Zealand, at three sampling zones (80–100 m.a.s.l; 120–140 m.a.s.l, 190–210 m.a.s.l) (Fig. 2). Recently-emerged adults of *Z*. *fenestrata* Clade 1 were collected from under stones in rapids or in the moss or vegetation next to the stream and immediately stored in absolute ethanol. Large nymphs were also collected from under stones in rapids and returned to the laboratory in a cooler, where they were reared in Styrofoam cups at 11 °C in water from their natal stream with small amounts of stream vegetation. Upon emerging as adults (within 30 days of sampling), individuals were immediately transferred to ethanol and stored at 4 °C. While the exact location was not identified for each sample, the approximate altitude was recorded within 20 m altitude. Samples were obtained from numerous different rocks across each sampling location.

### Morphological classification

**Figure 3:**
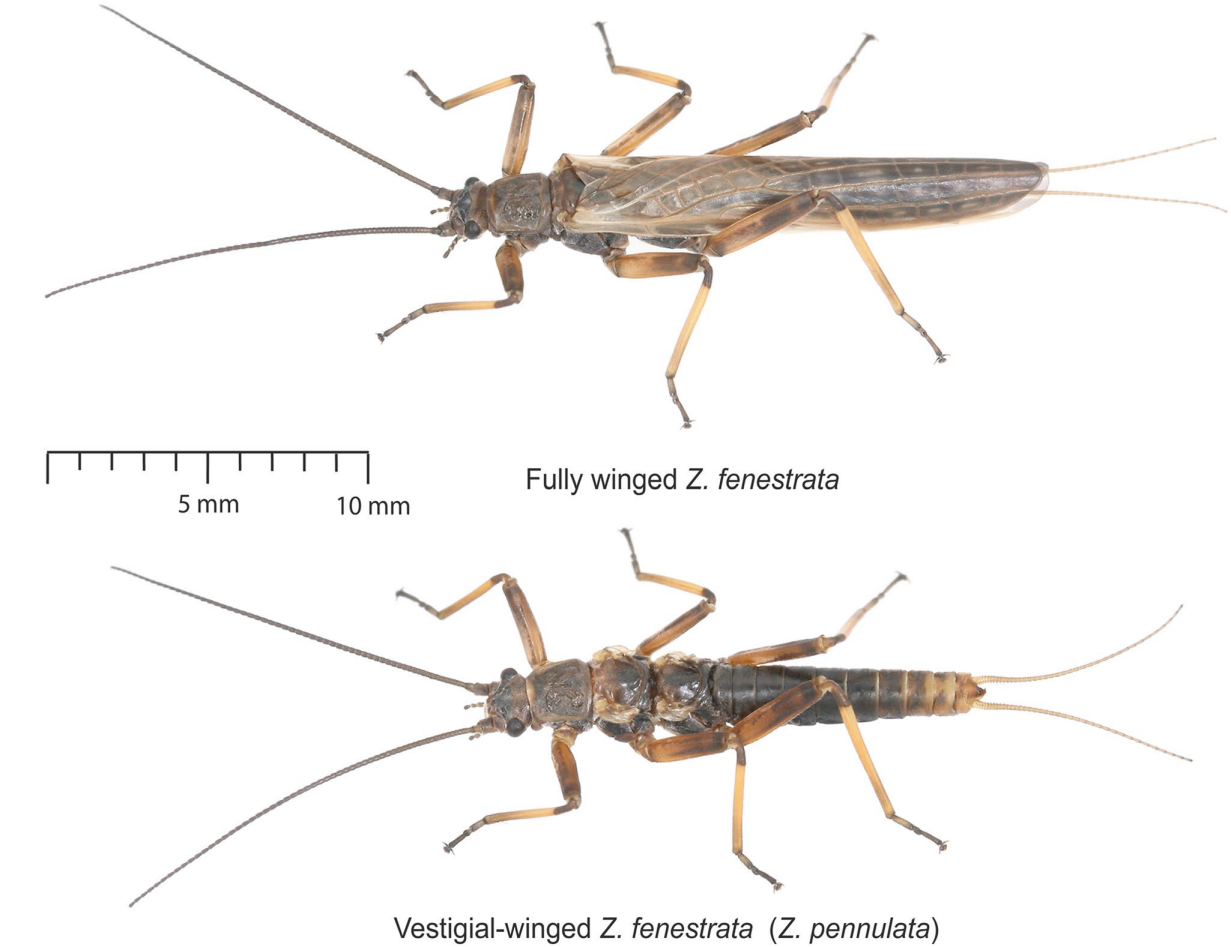
Distinct morphotypes of *Z*. *fenestrata* clade 1, showing relative wing lengths of the two forms.

All 127 individuals collected were photographed using a stereo microscope, and forewing length and body length were measured from a stage micrometer scale in ImageJ^75^. Forewings and hindwings are equally sized for each individual, therefore measuring both was not necessary. We visually sorted specimens into either a fully winged (macropterous) or vestigial-winged (micropterous) groups (Fig. 3). To examine the variation in wing length and body length we then visualized these data, and created a generalized linear model (GLM) for wing length based on body length, sex, sampling altitude and our previous wing length classification in R. These analyses tested for a clear pattern of wing dimorphism in this population, and to ensure the morphology classification was not biased by any additional influencing factors (e.g. size, altitude or sex).

### Dna extraction and sequencing

DNA extractions and GBS library prep were carried out for 96 individuals (34 fully winged, 62 vestigial-winged) using the same methodology as Dussex, *et al*.^64^. DNA extractions were carried out using DNeasy kits (Qiagen, Valencia, CA, USA) according to the manufacturer’s protocol using dissected head and femur tissue. Genotyping by sequencing library preparation followed the protocols of Elshire *et al*. (2011) with modifications as follows. DNA extractions were first dried using a vacuum centrifuge at 45 °C, then resuspended in 15 μL dH2O. To each sample, a uniquely barcoded PstI adapter was added (2.25 ng per sample; Morris *et al*. 2011). DNA digestion was performed using 4UPstI-HF (NewEngland Biolabs, Ipswich, MA; Morris *et al*. 2011) in 1X CutSmart BufferTM130 with incubation at 37 °C for 2 h. Adapters were ligated with T4 DNA ligase in 1X ligation buffer (New England Biolabs), followed by incubation at 16 °C for 90 min and 80 °C for 30 min. Purification was performed using a Qiagen MinElute PCR purification kit, with elution in 25 mL 1X TE. PCRs were carried out in 50 mL volumes containing 10 mL purified DNA, 1X MyTaqTM HS Master Mix (Bioline), and 1 mM each of PCR primers 5_AATGATACGGCGACCACCGAGATCTACACTCTTTCCCTACACGACGCTCTTCCGATC∗T and 5_ CAAGCAGAAGACGGCATACGAGATCGGTCTCGGCATTCCTGCTGAACCGCTCTTCCGATC∗T (where ∗ indicates phosphorothioation) as per Dussex *et al*.^64^. PCRs were run in a Mastercycler ep Gradient S (Eppendorf, Hamburg, Germany) under the following conditions: 72 °C for 5 min, 95 °C for 60 s, and 24 cycles of 95 °C for 30 s, 65 °C for 30 s, and 72 °C for 30 s, with a final extension step at 72 °C for 5 min. Sample concentrations were assessed using a NanoDrop spectrophotometer (Thermo Scientific) and all samples were pooled (20 ng DNA per sample). Size fractionation of the pooled library was achieved via electrophoresis on a 1.5% agarose gel, with a 300 bp size range from 200 to 500 bp selected for sequencing. A total of 96 samples were sequenced on one lane of an Illumina HiSeq 2500 using paired-end 75 bp reads.

### Analyses

#### Bioinformatic processing

All reads were trimmed, filtered and analyzed using the STACKS pipeline^76^ in order to create catalogues of comparable SNP loci. We optimized the pipeline according to the recommendations of Paris, *et al*.^77^. Initially, the PROCESS_RADTAGS module was used to separate reads by their barcode, remove low-quality reads (any read with an average Phred score <10 in any sliding window of 11 bp), trim all reads to 70 base pairs in length, and remove any reads that did not contain the enzyme recognition sequence. Next, the USTACKS module was used for the *de novo* assembly of raw reads into RAD tags. The minimum number of reads to create a stack was set at 3 (-m parameter in USTACKS), and the maximum number of pairwise differences between stacks was 2 (-M parameter in USTACKS). A catalogue of RAD tags was then generated using the 25 highest coverage individuals from each morphotype in CSTACKS. The distance allowed between catalogue loci (-n in CSTACKS) was increased to 2, after different trials were run to ensure loci were not inaccurately called as separate stacks. The execution of these components was accomplished using the STACKS denovo_map.pl script; in running this script, the optional -t flag was used to remove highly repetitive RAD tags during the USTACKS component of the pipeline. Following assembly and genotyping, the data were further filtered to maximize data quality. Using the POPULATIONS module, we retained only those loci that were genotyped in ≥50% of individuals and had a minor allele frequency ≥0.05 and a minimum stack depth of 10 (-m in POPULATIONS) for each individual. Genotypic data were exported from STACKS in GENEPOP format^78^ and converted for subsequent analyses using PGD SPIDER v. 2^79^.

#### Individual relatedness and population structure

We calculated an adjusted Genomic Relatedness Matrix (GRM)^80^ displaying this as a neighbor-joining relatedness tree. We investigated the number of populations (or clusters) represented in our data using FASTSTRUCTURE^81^ and the putatively neutral SNP dataset, default parameters, a logistic prior, and *K* from 1 to 6. The appropriate number of model components that explained structure in the dataset was determined using the *chooseK*.*py* function^81^. Results for the identified optimal values of *K* were visualized using DISTRUCT^82^. We also estimated the number of clusters using the *find*.*clusters* command in ADEGENET, with optimization based on the Bayesian Information Criterion (BIC). Finally, we also calculated expected and observed heterozygosity using Arlequin 3.5^83^.

#### Outlier loci detection and annotation

Due to the limitations of differentiation-based methods and the potentially high false positive rates when looking for outlier loci under divergent selection^84,85^, we utilized two distinct approaches: 1) an *F*_*ST*_ based outlier approach between *a priori* morphotype-pairs implemented in BAYESCAN^86^ and 2) a hierarchical Bayesian modeling approach implemented in PCADAPT^87^.

BAYESCAN analyses can give spurious results when there is significant over-representation of one of the groups being compared^88^. Due to the sample size of vestigial-winged specimens being approximately twice that of fully winged specimens, we performed two independent BAYESCAN runs, both including all fully winged individuals, but each with a different half of the vestigial-winged group. These two comparisons therefore each had a balanced design, and can be used to evaluate the generality of outlier loci detected across partially independent comparisons (given that one comparison group remains the same while the other changes). For each analysis, BAYESCAN was run using 10,000 output iterations, a thinning interval of 10, 20 pilot runs of length 10,000, and a burn-in period of 10,000, with prior odds of the neutral model of 10. We recorded all loci with a q-value of 0.2 or less, which equates to a false discovery rate of 20%. Q-values are far more stringent than p-values in classical statistics as they are adjusted for the false discovery rate given multiple comparisons, rather than the individual false positive rates in each comparison^89^. To better understand the rates of false positive identification for outlier loci in this dataset, we also undertook 20 runs of BAYESCAN using identical parameters but comparing randomized groups of individuals (each also consisting of 34 individuals).

We also conducted outlier detection as implemented in PCADAPT^87^. The number of Principal Components retained (*K*) for each analysis was determined by the graphical approach based on the scree-plot^90^, as recommended by Luu, *et al*.^87^.

### Ethical statement

All experiments were performed in accordance with University of Otago ethics committee regulations and guidelines.

## Results

### Morphology

**Figure 4:**
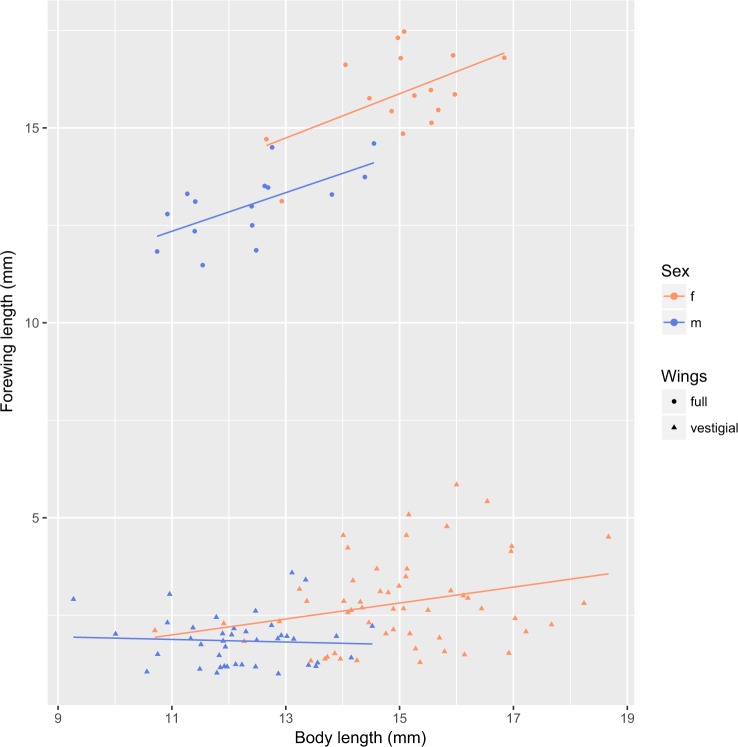
Variation in the relative wing length and body length of *Z*. *fenestrata* Clade 1 from Black Jacks Creek.

Of 127 adults measured in this *Z*. *fenestrata* Clade 1 population, we found clear wing dimorphism for both males and females, with an approximately even number of each sex sampled (Figs 3, 4). Fully winged individuals had an average forewing length: body length ratio of 1.06 ± 0.15 (SE), while the vestigial-winged individuals had an average forewing length: body length ratio of 0.26 ± 0.28 (SE), and there was no overlap in the distribution of wing lengths between groups, thus clearly defining the two groups morphologically. This difference in wing length was highly significant (t = −57.479, p < 2e-16). Sampling altitude (over this small altitudinal range) had no significant effect on the proportion of each morphotype, nor did it affect body length or wing length. Sex was significantly correlated with forewing length (t = −3.331, p = 0.00114), with females consistently having both longer forewings and bodies than males for both the fully winged and vestigial-winged forms, and there was also a significant positive correlation between body length and wing length within each sex (t = 2.811, p = 0.00575).

### GBS genotypic data and alignment

We obtained a total of 182,364,061 reads, with 162,960,471 retained after quality filtering. Each individual had on average 1,810,615 reads (s.d. = 300,690) with an average depth per tag of 3.1× . Following GBS, processing and filtering, we collected genotypic data at 6,843 SNPs across 96 of the measured 127 *Z*. *fenestrata* Clade 1 individuals – leaving out randomly selected vestigial-winged individuals, because this dataset was far larger than the fully winged dataset. We only retained one SNP per tag, with the sequences of tags provided in Supplementary Table 1. Observed heterozygosity (mean = 0.29, s.d. = 0.25) did not differ significantly from expected heterozygosity (mean −0.26, s.d. = 0.15).

**Figure 5:**
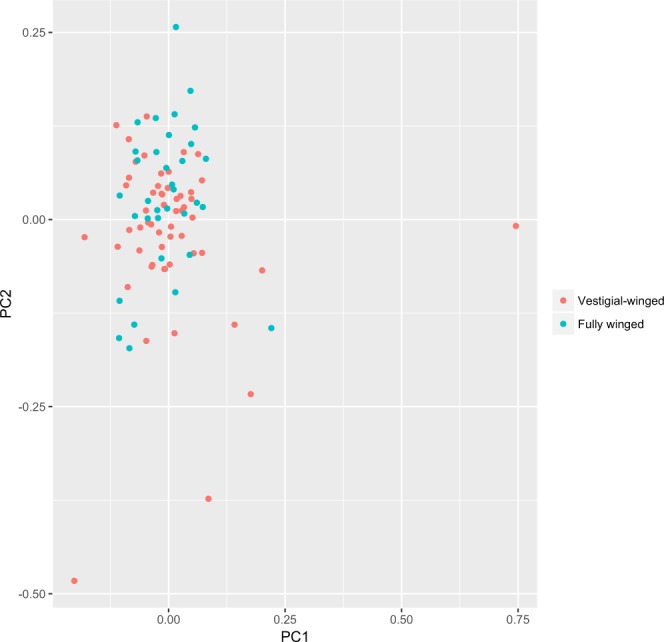
Principal component analysis of *Z*. *fenestrata* Clade 1 genetic differentiation in Black Jacks Creek.

We detected no genome-wide population differentiation among the sample groups, irrespective of the analytical approach implemented. First, FASTSTRUCTURE indicated an optimal number of clusters as 1, and when higher number of clusters were investigated, no clear pattern of differentiation emerged. Secondly, using the *find*.*clusters* function in ADEGENET, the optimal number of clusters was 1, and no trend in differential clustering was visible for higher values of K. Third, no structure was evident in the principal component analyses of genetic differentiation (Fig. 5), and no pattern of relatedness linked to wing morphology was observed (Supplementary Fig. 1).

Given these results, we conclude that there is no neutral population structure between fully winged and vestigial-winged individuals when sampled from the same location, and no differentiation among sampling localities (i.e. there is one homogeneous population over the sampling range). Given this apparent panmixia, genetic differences associated with morphotype differentiation, if present, must therefore be limited to small regions of the genome, likely indicating loci under divergent selection.

### Outlier loci detection and comparison

Given that no principal components correlated to morphotype differentiation, PCADAPT was unable to detect outliers associated with morphotypes, instead only identifying loci associated with the differentiation of a handful of slightly divergent individuals (Fig. 5).

Given that the data set included 34 fully winged individuals compared with 62 vestigial-winged individuals, we conducted two separate BAYESCAN analyses, dividing the vestigial-winged population sample in two. This approach was adopted to avoid the effects of uneven sample sizes, which can disproportionately skew outlier detection results^88^. These analyses also provided an opportunity to identify any loci significantly differentiated across both of these largely independent comparisons.

**Figure 6:**
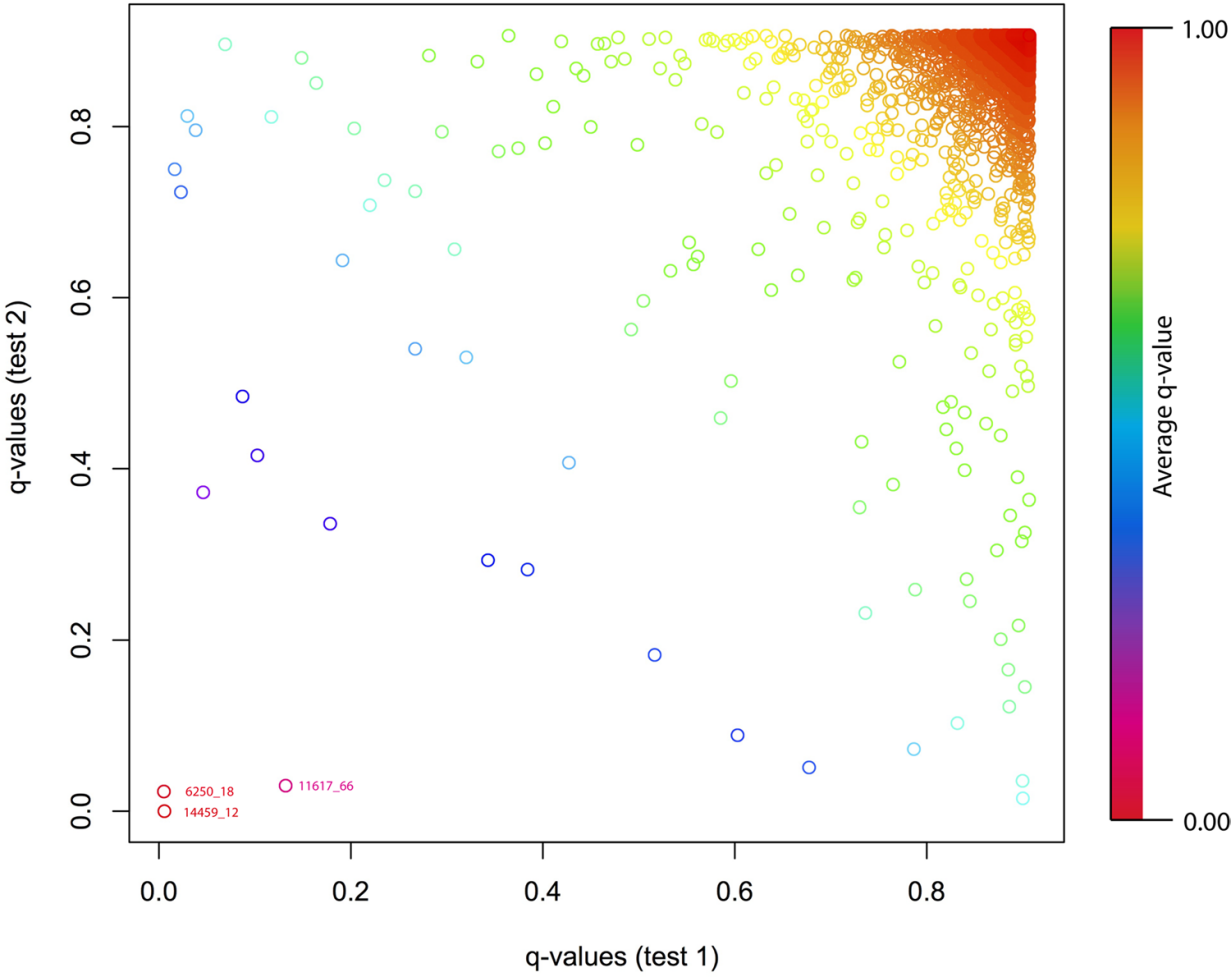
Scatterplot comparing the q-values obtained from the two independent BAYESCAN comparisons of fully winged and vestigial-winged morphotypes of *Z*. *fenestrata* Clade 1 sampled in Black Jacks Creek.

The two BAYESCAN runs detected 17 and 14 outlier loci with q-values < 0.2, 9 and 8 with q-values < 0.1, and 7 and 5 with q-values < 0.05 (Supplementary Table 2). Of these outlier loci, three were identified in both runs, with one locus (14459_12) identified as the most significantly differentiated SNP for both comparisons, with q-values of (0.00570 and <0.00000) (Fig. 6). In independent comparisons involving random differences between groups, one would expect 0.03 loci to be detected as outliers in both comparisons, and the probability that the most differentiated locus would be identical would be extremely low (e.g. 1/6843)^2^.

In the randomized BAYESCAN runs, an average of 10.6 outlier loci were detected at q-values of <0.2, with a maximum of 13 outlier loci detected. This number of outlier loci is lower than that generated by our winged vs. wingless analyses, although not greatly, suggesting that many of the recorded outliers are likely to be false positives. However, the minimum q-value recorded across these random comparisons was 0.026, substantially higher than the lowest values detected in our real fully winged vs. vestigial winged analyses. These findings thus provide evidence that the strongest outliers detected in our analysis are truly associated with phenotypic variation, rather than representing false positives.

The observed differentiation between fully winged and vestigial-winged individuals at these outlier loci suggests that there are regions of the genome highly differentiated between these two morphotypes. Due to the paucity of genomic data published for Plecoptera, we were unable to map these outlier loci via BLAST-n to genomic regions to identify the genes present in the surrounding regions.

## Discussion

In this study, we tested for a genetic basis for wing reduction in the New Zealand stonefly *Z*. *fenestrata* Clade 1. While we found no neutral population structure among the two sympatric morphotypes, we detected outlier loci between fully winged and vestigial-winged *Z*. *fenestrata* Clade 1 individuals, with several of the most highly differentiated outlier loci common to distinct sample comparisons. These results match the predictions of a ‘divergence with gene flow’ scenario, where small regions of the genome (genomic islands of divergence) are highly differentiated, contrasting with lower differentiation across the rest of the genome^91–93^. These results strongly support the hypothesis that wing reduction in *Z*. *fenestrata* Clade 1 is at least partially genetically determined, and not solely an environmentally determined polyphenism. Under such as scenario it is unsurprising we were unable to detect differentiation between morphotypes using analyses such as FASTSTRUCTURE and ADEGENET, as the proportion of SNPs differentiating morphotypes is small. In a similar system where a few SNPs are linked to the causal mechanisms behind ecotype divergence in sockeye salmon, no population differentiation was detectable among a complete marker set in several lake systems, despite almost fixed differences between ecotypes at these outlier SNPs^94,95^.

The current study used a relatively low threshold (<0.2) for reporting q-values for outlier loci, although this does not mean that *all* of these loci are necessarily true outliers in this population. Indeed, outlier loci should only ever be treated as hypotheses that require further lines of evidence for validation, ideally using independent datasets. In this study, we considered outlier loci from individual runs to be ‘hypotheses’, and then tested these hypotheses on the other independent dataset, under the assumption that loci genuinely linked to wing phenotypic divergence would be detected in both tests. Our comparisons of winged versus wing-reduced samples revealed four highly significant outlier loci, each with q-values substantially lower than any values obtained from randomised runs. Additionally, the fact that three of these outliers were detected across multiple independent runs increases confidence that they represent real genetic differences strongly associated with *Z*. *fenestrata* wing variation.

Our study has identified several SNPs potentially associated with *Zelandoperla* phenotypic variation, although we do not infer that these SNPs themselves have any direct causal relationship with the distinct wing morphotypes. Rather, these SNPs are likely to be in linkage with changes in nearby regions of the genome that influence morphotype^96^. As regions linked to the genetic changes underlying phenotypic differences can be very large^97,98^, we would require a well-annotated and near-complete genomic sequence before we could speculate as to the specific changes responsible for wing polymorphism. Currently, the genomic resources for *Z*. *fenestrata* (and all Plecoptera) are too incomplete to allow us to assess if the outlier loci identified in our analyses are adjacent to each other, or more generally, if they are in islands of divergence. Without these genomic resources, it is also impossible to speculate as to the potential underlying genes that may be responsible for these two phenotypes. With further work creating a genome assembly for this species, we will be able to look at the specific genomic regions linked to the outlier SNPs defined in this study.

Given a probable genetic basis for wing morphotype, and evidence for divergent selection for different morphotypes at different altitudes as indicated by the broader altitudinal distribution of the two morphotypes^32,64^, this system is potentially an example of early ecological divergence with gene flow, similar to recent examples of ecological speciation e.g.^99,100^. While reproductive barriers do not apparently exist between these two sympatric morphotypes in Clade 1, the broad system we describe demonstrates the effects of divergent selection at different altitudes, with ongoing gene flow where the two forms meet. A similar system of repeated phenotypic divergence between high and low altitude insect morphotypes (with some wing-reduced forms) has been seen in hunter beetles in the Galapagos Islands^101^. From both the present study and previous work, there appears to be minimal neutral divergence (beyond possible isolation by distance)^64^ between fully winged and vestigial-winged morphotypes within the same stream. This pattern may indicate that the underlying genetic basis for wing loss is simple, comparable to the mechanisms underlying melanism in various insects^102–104^. If this is the case, then instead of a ‘divergence with gene flow’ scenario^105^, we may simply be observing the effects of two alleles that lead to different phenotypes, with differential selection across distinct environments, similar to industrial melanism in the peppered moth^104^.

When populations occupy different habitats, divergent natural selection can cause differentiation in ecologically important characters (for review, see Schluter^106^), and conversely, gene flow between divergent populations acts as a homogenizing force, eroding population differentiation^107^. In the majority of *Z*. *fenestrata* Clade 1 populations, vestigial-winged populations occupy higher altitudes and are largely allopatric to the lower altitude fully winged populations. It appears that gene flow over any distance is extremely low for *Z*. *fenestrata*, as evidenced by the fine-scale genetic structure between nearby streams^64^. This poor flighted dispersal ability may contribute towards maintaining the spatial divergence between morphotypes often observed, despite the observed homogenization across the majority of the genome in geographic regions of population overlap.

Based on evidence from the current study, we infer a likely genetic contribution to the wing phenotypic variation observed in *Zelandoperla*. However, it remains possible that an environmental component may also contribute to this morphological differentiation. Indeed, in other insect lineages, the penetrance of genetic factors regulating wing development can be mediated by environmental factors, and therefore the expression of phenotype can be highly complex^73,74^. The differing patterns of wing loss observed in different populations of *Z*. *fenestrata* Clade 1 could potentially indicate interacting roles played between the environment and genetics. While it remains possible that environmentally determined gene expression could be partially responsible for wing variation across the *Z*. *fenestrata* species group, additional research is required to address this speculative suggestion.

Untangling the precise mechanisms of wing reduction in the *Z*. *fenestrata* species group, including testing for an environmentally induced component to these alternative developmental pathways, will require further experimentation. While the *Z*. *fenestrata* species group is a fascinating system to study the mechanisms of wing reduction in insects, the group does have some life-history and population characteristics that create challenges for understanding the mechanism(s) behind wing loss. *Z*. *fenestrata* can have a long generation time (perhaps involving years as a wingless nymph), making breeding experiments and QTL studies challenging. Furthermore, their habitat is fast flowing rapids in highly oxygenated streams with cold water, making them difficult to raise in laboratory settings for a full life cycle, and hindering reciprocal translocation experiments in the wild. Studies of gene expression in the developing notums of *Z*. *fenestrata* nymphs of different morphotypes should provide more information to the regulatory mechanisms and pathways underlying wing development in this species.

Our results reinforce the need for taxonomic revision for this species group, as there is no genetic evidence for the separation of vestigial-winged morphotypes of *Z*. *fenestrata* Clade 1 into the separate taxon *Z*. *pennulata*. Along with there being no neutral genetic differentiation between co-occurring morphotypes of this species, we found no temporal or spatial segregation of the two morphotypes: recently-emerged fully winged and vestigial-winged individuals were collected simultaneously, and in immediate sympatry. These results are consistent with the completely overlapping temporal patterns of emergence documented by McLellan^63^. While fully winged and vestigial-winged morphotypes within *Z*. *fenestrata* Clade 1 appear to have minimal genetic differentiation between them, suggesting they are conspecific, the species status of this clade has not been formally reviewed. The remaining clades of the *Z. fenestrata* species group appear to have different propensities for wing reduction (Clade 1 in alpine environments, Clade 2–4 appear to lack wing reduction, while Clade 5 is always wing-reduced), future studies will aim to assess genetic variation at potential wing-loss loci across the entire group.

## Conclusion

Wing dimorphism is a common trait across many species of stoneflies, but the mechanisms behind this phenotypic diversity have yet to be investigated. *Zelandoperla fenestrata* Clade 1 presents an ideal taxon to examine this phenomenon, potentially revealing the generalized mechanisms behind wing reduction in this order. Our results for this spatially overlapping population of fully winged and vestigial-winged *Z*. *fenestrata* Clade 1 morphotypes support the hypothesis that wing development has a genetic mechanism rather than being solely environmentally determined. While there was no neutral genetic structure between wing morphotypes, outlier loci were identified between these two groups. While it is possible that these outlier loci are not themselves linked with the specific causative changes associated with wing development, any genetic differences linked to wing morphotype differentiation in an otherwise sympatric population must indicate that there is some genetic differentiation between morphotypes. Further examination of these outlier loci may reveal the underlying genes linked to wing reduction in this species.

## Electronic supplementary material

Supplementary Figure 1

Supplementary Table 1

Supplementary Table 2

Supplementary Information

## Data Availability

All processed data from Stacks is included in the supplementary information.
