# Supplementary material for: Genotyping-by-sequencing supports a genetic basis for wing reduction in an alpine New Zealand stonefly
Source: Sci Rep. 2018 Nov 2;8:16275. doi: 10.1038/s41598-018-34123-1 (PMC6215011; doi:10.1038/s41598-018-34123-1)
Supplement: Supplementary file 1 — Supplementary Figure 1 [file 41598_2018_34123_MOESM1_ESM.pdf]

Genotyping-by-sequencing supports a genetic basis for wing reduction in an alpine New Zealand stonefly.

Andrew J. Veale[1,2], Brodie J. Foster[1], Peter K. Dearden[3], Jonathan M. Waters[1]
1. Department of Zoology, University of Otago, Dunedin 9016, New Zealand
2. Department of Environmental and Animal Sciences, Unitec, Auckland 1025, New Zealand
3. Department of Biochemistry, University of Otago, Dunedin 9016, New Zealand

Supplementary Material.

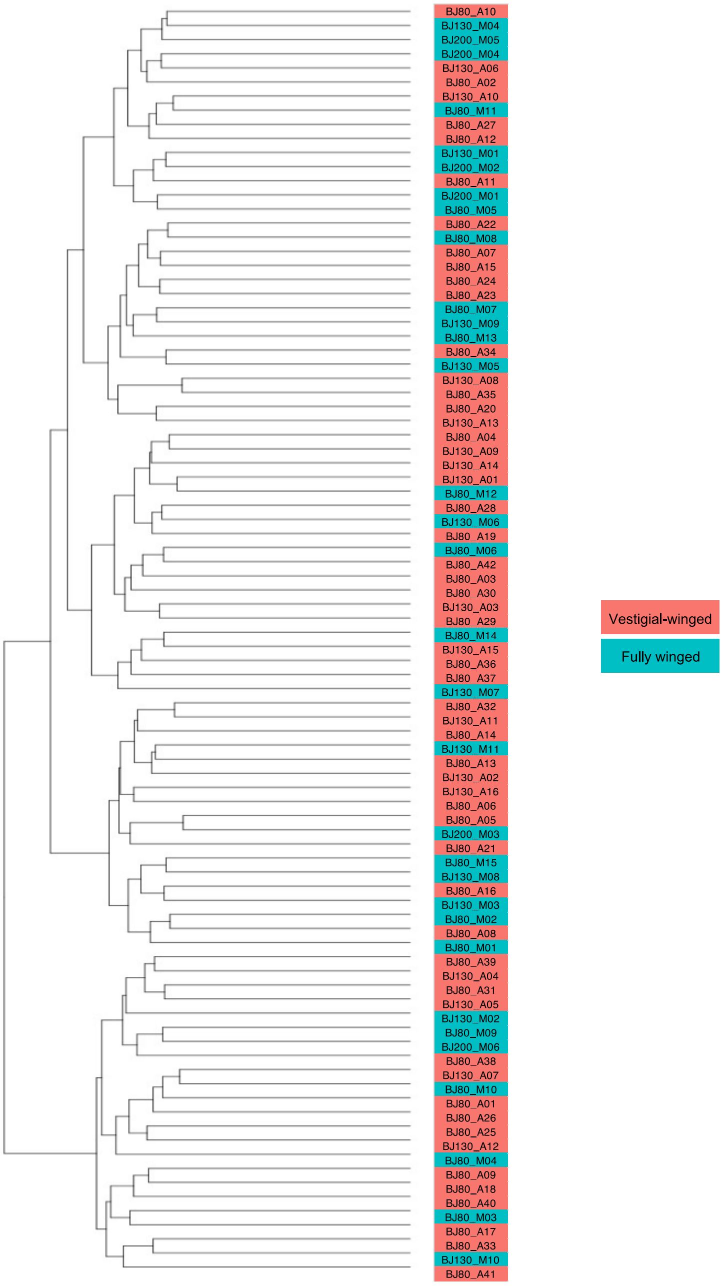

Supplementary Figure 1. Relatedness tree of Z. fenestrata Clade 1 samples showing the lack of phylogenetic differentiation between wing morphologies.
